# (2*Z*,3*Z*)-3,4-Dihydro-2*H*-1,4-benzothia­zine-2,3-dione dioxime dihydrate

**DOI:** 10.1107/S1600536808023301

**Published:** 2008-07-31

**Authors:** Ali Kakanejadifard, Vahid Amani

**Affiliations:** a Department of Chemistry, Faculty of Science, Lorestan University, Khorramabad, Iran; b Department of Chemistry, Islamic Azad University, Shahr-e-Rey Branch, Tehran, Iran

## Abstract

In the mol­ecule of the title compound, C_8_H_11_N_3_O_4_S, the thia­zine ring adopts an envelope conformation. In the crystal structure, inter­molecular N—H⋯O, O—H⋯O and O—H⋯N hydrogen bonds link the mol­ecules.

## Related literature

For related literature, see: Kakanejadifard, Niknam *et al.* (2007 ▶); Kakanejadifard, Saniei *et al.* (2007 ▶); Kakanejadifard & Niknam (2006 ▶); Kakanejadifard & Amani (2008 ▶). For general background, see: Jones *et al.* (1961 ▶); Schrauzer & Kohnle (1964 ▶); Yari *et al.* (2006 ▶); Hashemi *et al.* (2006 ▶); Ghiasvand *et al.* (2004 ▶, 2005 ▶); Kakanejadifard, Niknam & Zabardasti (2007 ▶); Gok & Kantekin (1997 ▶); Hughes (1981 ▶).
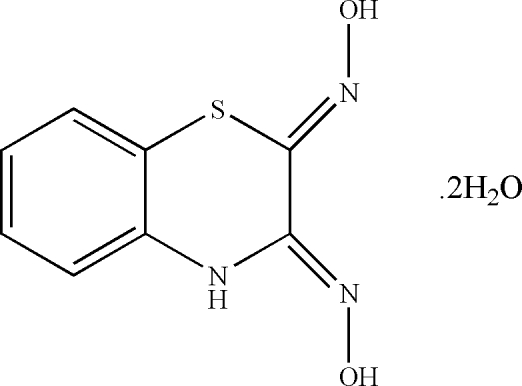


## Experimental

### 

#### Crystal data

C_8_H_7_N_3_O_2_S·2H_2_O
*M* *_r_* = 245.26
Orthorhombic, 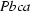

*a* = 9.1636 (18) Å
*b* = 9.8195 (18) Å
*c* = 24.165 (4) Å
*V* = 2174.4 (7) Å^3^
*Z* = 8
Mo *K*α radiation
μ = 0.30 mm^−1^
*T* = 120 (2) K
0.5 × 0.5 × 0.1 mm

#### Data collection

Bruker SMART 1000 CCD area-detector diffractometer
Absorption correction: multi-scan (*SADABS*; Sheldrick, 1998 ▶) *T*_min_ = 0.859, *T*_max_ = 0.974
15018 measured reflections
2337 independent reflections
1482 reflections with *I* > 2σ(*I*)
*R*_int_ = 0.082

#### Refinement

*R*[*F* ^2^ > 2σ(*F* ^2^)] = 0.046
*wR*(*F* ^2^) = 0.107
*S* = 1.02
2337 reflections
145 parameters
H-atom parameters constrained
Δρ_max_ = 0.43 e Å^−3^
Δρ_min_ = −0.25 e Å^−3^

### 

Data collection: *SMART* (Bruker, 1998 ▶); cell refinement: *SAINT-Plus* (Bruker, 1998 ▶); data reduction: *SAINT-Plus*; program(s) used to solve structure: *SHELXTL* (Sheldrick, 2008 ▶); program(s) used to refine structure: *SHELXTL*; molecular graphics: *SHELXTL*; software used to prepare material for publication: *SHELXTL*.

## Supplementary Material

Crystal structure: contains datablocks I, global. DOI: 10.1107/S1600536808023301/hk2502sup1.cif

Structure factors: contains datablocks I. DOI: 10.1107/S1600536808023301/hk2502Isup2.hkl

Additional supplementary materials:  crystallographic information; 3D view; checkCIF report

## Figures and Tables

**Table 1:** Hydrogen-bond geometry (Å, °)

*D*—H⋯*A*	*D*—H	H⋯*A*	*D*⋯*A*	*D*—H⋯*A*
O1*W*—H1*WA*⋯N11^i^	0.85	2.11	2.931 (2)	162
O1*W*—H1*WA*⋯N13^i^	0.85	2.62	3.225 (2)	129
O1*W*—H1*WB*⋯O2*W*^i^	0.85	2.00	2.845 (2)	171
O2*W*—H2*WA*⋯N13^ii^	0.85	2.02	2.853 (3)	168
N4—H4⋯O14	0.87	2.08	2.493 (2)	108
O2*W*—H2*WB*⋯O1*W*	0.85	1.94	2.780 (2)	171
O12—H12⋯O2*W*^iii^	0.82	1.89	2.699 (2)	171
O14—H14⋯O1*W*	0.82	1.85	2.673 (2)	179

Symmetry codes: (i) 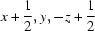; (ii) 
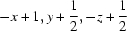
; (iii) 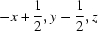.

**Fig. 1.**
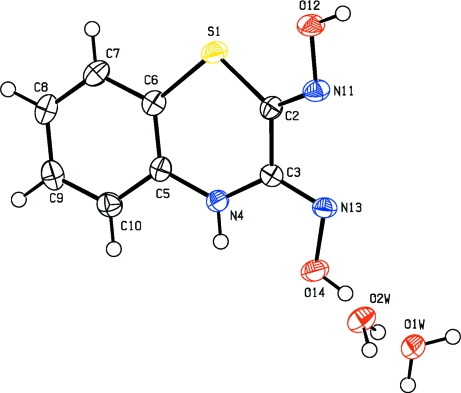
The molecular structure of the title molecule, with the atom-numbering scheme. Displacement ellipsoids are drawn at the 50% probability level.

**Fig. 2.**
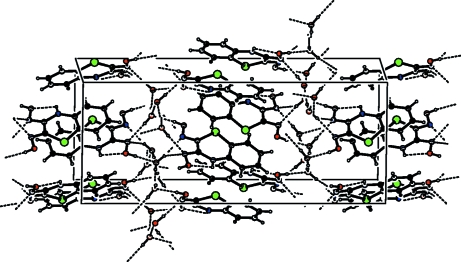
A packing diagram of the title molecule. Hydrogen bonds are shown as dashed lines.

## supplementary crystallographic information

### Comment

We have been interested in the synthesis and chemical behavior of vic-dioximes, in the past decade. In our investigations, the reaction of amines with dichloroglyoxime or cyanogendi-N-oxide resulted in various symmetrically substituted diaminoglyoxime derivatives, in which some of them were quite suitable to act, as donor species, towards some transition metal ions (Kakanejadifard, Niknam *et al.*, 2007; Kakanejadifard, Saniei *et al.*, 2007; Kakanejadifard & Niknam, 2006; Kakanejadifard & Amani, 2008). Some oximes are widely used for various purposes in organic, inorganic, bioinorganic, pigment, analytical, dyes and medical chemistry (Jones *et al.*, 1961; Schrauzer & Kohnle, 1964; Yari *et al.*, 2006; Hashemi *et al.*, 2006; Ghiasvand *et al.*, 2004; Ghiasvand *et al.*, 2005; Kakanejadifard, Niknam & Zabardasti, 2007). vic-Dioximes, containing mildly acidic hydroxyl groups and slightly basic nitrogen atoms, are amphoteric and their transition metal complexes have been widely investigated as analytical reagents (Gok & Kantekin, 1997), and models for biological systems such as vitamin B_12_ (Hughes, 1981). We report herein the synthesis and crystal structure of the title compound.

In the molecule of the title compound (Fig. 1), the bond lengths and angles are within normal ranges. Ring A (S1/N4/C2/C3/C5/C6) adopts envelope conformation, with C2 atom displaced by 0.248 (3) Å from the plane of the other ring atoms. Ring B (C5–C10) is, of course, planar.

In the crystal structure, intermolecular N—H···O, O—H···O and O—H···N hydrogen bonds (Table 1) link the molecules (Fig. 2), in which they may be effective in the stabilization of the structure.

### Experimental

For the preparation of the title compound, a solution of NaHCO_3_ (0.05 g, 0.6 mmol) in aqueous EtOH (10 ml) was added to a magnetically stirred solution of dicholoroglyoxime (1.57 g, 10 mmol) in aqueous EtOH (15 ml) and a solution of 2-aminothiophenole (1.25 g, 10 mmol) in EtOH (15 ml) at room temperature. The solution was stirred for 4 h and then the mixture was filtered. The filtrate was placed at room temperature for 24 h. The gray precipitate was removed by filtration and precipitate was washed with cold THF. It was recrystallized from 2-propanol in one week (yield; 0.31 g, 74.2%, m.p. 492 K).

### Refinement

H atoms were positioned geometrically, with O—H = 0.82 Å (for OH), N—H = 0.8691 Å (for NH), O—H = 0.8499–0.8501 Å (for H_2_O) and C—H = 0.93 Å for aromatic H, and constrained to ride on their parent atoms with U_iso_(H) = xU_eq_(C,N,O), where x = 1.5 for OH H, x = 0.95 for NH H, and x = 1.2 for all other H atoms.

### Crystal data

**Table:**

C_8_H_7_N_3_O_2_S·2H_2_O	*F*_000_ = 1024
*M**_r_* = 245.26	*D*_x_ = 1.498 Mg m^−^^3^
Orthorhombic, *P**b**c**a*	Mo *K*α radiation λ = 0.71073 Å
Hall symbol: -P 2ac 2ab	Cell parameters from 910 reflections
*a* = 9.1636 (18) Å	θ = 2.5–26.3º
*b* = 9.8195 (18) Å	µ = 0.30 mm^−^^1^
*c* = 24.165 (4) Å	*T* = 120 (2) K
*V* = 2174.4 (7) Å^3^	Plate, yellow
*Z* = 8	0.5 × 0.5 × 0.1 mm

### Data collection

**Table:**

Bruker SMART 1000 CCD area-detector diffractometer	2337 independent reflections
Radiation source: fine-focus sealed tube	1482 reflections with *I* > 2σ(*I*)
Monochromator: graphite	*R*_int_ = 0.082
*T* = 120(2) K	θ_max_ = 27.0º
φ and ω scans	θ_min_ = 1.7º
Absorption correction: multi-scan(SADABS; Sheldrick, 1998)	*h* = −11→11
*T*_min_ = 0.859, *T*_max_ = 0.974	*k* = −12→12
15018 measured reflections	*l* = −30→28

### Refinement

**Table:**

Refinement on *F*^2^	Secondary atom site location: difference Fourier map
Least-squares matrix: full	Hydrogen site location: difference Fourier map
*R*[*F*^2^ > 2σ(*F*^2^)] = 0.046	H-atom parameters constrained
*wR*(*F*^2^) = 0.107	*w* = 1/[σ^2^(*F*_o_^2^) + (0.0534*P*)^2^ + 0.01*P*] where *P* = (*F*_o_^2^ + 2*F*_c_^2^)/3
*S* = 1.02	(Δ/σ)_max_ < 0.001
2337 reflections	Δρ_max_ = 0.43 e Å^−^^3^
145 parameters	Δρ_min_ = −0.25 e Å^−^^3^
Primary atom site location: structure-invariant direct methods	Extinction correction: none

### Special details

**Table:**

Geometry. All e.s.d.'s (except the e.s.d. in the dihedral angle between two l.s. planes) are estimated using the full covariance matrix. The cell e.s.d.'s are taken into account individually in the estimation of e.s.d.'s in distances, angles and torsion angles; correlations between e.s.d.'s in cell parameters are only used when they are defined by crystal symmetry. An approximate (isotropic) treatment of cell e.s.d.'s is used for estimating e.s.d.'s involving l.s. planes.
Refinement. Refinement of F^2^ against ALL reflections. The weighted R-factor wR and goodness of fit S are based on F^2^, conventional R-factors R are based on F, with F set to zero for negative F^2^. The threshold expression of F^2^ > 2sigma(F^2^) is used only for calculating R-factors(gt) etc. and is not relevant to the choice of reflections for refinement. R-factors based on F^2^ are statistically about twice as large as those based on F, and R- factors based on ALL data will be even larger.

### Fractional atomic coordinates and isotropic or equivalent isotropic displacement parameters (Å^2^)

**Table:**

	*x*	*y*	*z*	*U*_iso_*/*U*_eq_	
S1	0.18557 (6)	0.97098 (6)	0.44569 (2)	0.02751 (18)	
O1W	0.74924 (16)	1.21073 (15)	0.24961 (6)	0.0294 (4)	
H1WA	0.7567	1.1544	0.2230	0.035*	
H1WB	0.8298	1.2523	0.2539	0.035*	
O2W	0.50348 (16)	1.37490 (17)	0.23976 (6)	0.0302 (4)	
H2WA	0.5201	1.4420	0.2185	0.036*	
H2WB	0.5838	1.3317	0.2411	0.036*	
O12	0.07401 (16)	0.93917 (17)	0.34431 (6)	0.0326 (4)	
H12	0.0423	0.9158	0.3140	0.049*	
O14	0.61786 (17)	1.16110 (16)	0.34627 (6)	0.0316 (4)	
H14	0.6570	1.1757	0.3163	0.047*	
N4	0.4660 (2)	1.14780 (19)	0.43170 (7)	0.0231 (4)	
H4	0.5489	1.1895	0.4276	0.022*	
N11	0.20659 (19)	1.00628 (19)	0.33728 (8)	0.0268 (5)	
N13	0.48394 (19)	1.09374 (19)	0.33772 (8)	0.0236 (4)	
C2	0.2680 (2)	1.0265 (2)	0.38436 (9)	0.0223 (5)	
C3	0.4132 (2)	1.0922 (2)	0.38433 (9)	0.0211 (5)	
C5	0.4075 (2)	1.1389 (2)	0.48497 (9)	0.0212 (5)	
C6	0.2847 (2)	1.0617 (2)	0.49653 (9)	0.0228 (5)	
C7	0.2320 (2)	1.0540 (2)	0.55071 (9)	0.0269 (5)	
H7	0.1500	1.0014	0.5584	0.032*	
C8	0.3006 (3)	1.1236 (2)	0.59269 (9)	0.0289 (6)	
H8	0.2651	1.1183	0.6287	0.035*	
C9	0.4222 (3)	1.2015 (2)	0.58121 (10)	0.0302 (6)	
H9	0.4683	1.2493	0.6094	0.036*	
C10	0.4756 (3)	1.2084 (2)	0.52783 (9)	0.0269 (6)	
H10	0.5583	1.2602	0.5205	0.032*	

### Atomic displacement parameters (Å^2^)

**Table:**

	*U*^11^	*U*^22^	*U*^33^	*U*^12^	*U*^13^	*U*^23^
S1	0.0211 (3)	0.0357 (3)	0.0257 (3)	−0.0062 (3)	0.0008 (2)	0.0020 (3)
O1W	0.0243 (9)	0.0344 (9)	0.0295 (9)	−0.0021 (7)	0.0050 (7)	−0.0041 (8)
O2W	0.0251 (8)	0.0325 (9)	0.0329 (10)	0.0035 (7)	0.0048 (7)	0.0067 (8)
O12	0.0237 (9)	0.0466 (11)	0.0275 (9)	−0.0140 (8)	−0.0027 (7)	−0.0017 (8)
O14	0.0269 (9)	0.0398 (10)	0.0282 (9)	−0.0107 (8)	0.0022 (7)	0.0030 (8)
N4	0.0217 (9)	0.0262 (11)	0.0213 (10)	−0.0026 (8)	0.0009 (8)	−0.0002 (8)
N11	0.0201 (10)	0.0316 (11)	0.0287 (11)	−0.0038 (8)	−0.0016 (8)	0.0025 (9)
N13	0.0209 (10)	0.0249 (10)	0.0251 (10)	−0.0049 (8)	−0.0011 (8)	0.0008 (8)
C2	0.0200 (11)	0.0249 (11)	0.0220 (12)	0.0024 (10)	0.0004 (9)	0.0010 (10)
C3	0.0215 (11)	0.0200 (11)	0.0219 (12)	0.0014 (10)	−0.0025 (9)	0.0018 (10)
C5	0.0220 (11)	0.0198 (11)	0.0218 (12)	0.0067 (10)	0.0003 (9)	0.0017 (10)
C6	0.0210 (12)	0.0239 (12)	0.0236 (13)	0.0061 (9)	−0.0015 (9)	0.0033 (9)
C7	0.0224 (11)	0.0316 (14)	0.0268 (13)	0.0050 (10)	0.0023 (10)	0.0060 (11)
C8	0.0320 (13)	0.0327 (13)	0.0219 (13)	0.0095 (11)	0.0042 (11)	0.0021 (11)
C9	0.0413 (15)	0.0251 (13)	0.0243 (14)	0.0050 (11)	−0.0033 (11)	−0.0024 (10)
C10	0.0310 (13)	0.0209 (12)	0.0289 (14)	0.0006 (10)	−0.0003 (11)	0.0019 (10)

### Geometric parameters (Å, °)

**Table:**

S1—C2	1.750 (2)	C2—C3	1.479 (3)
S1—C6	1.769 (2)	C5—C6	1.385 (3)
S1—C5	2.785 (2)	C5—C10	1.388 (3)
S1—C3	2.823 (2)	C6—C7	1.398 (3)
O12—N11	1.393 (2)	C7—C8	1.376 (3)
O12—H12	0.8200	C7—H7	0.9300
O14—N13	1.409 (2)	C8—C9	1.380 (3)
O14—C3	2.195 (3)	C8—H8	0.9300
O14—H14	0.8200	C9—C10	1.381 (3)
N4—C3	1.357 (3)	C9—H9	0.9300
N4—C5	1.397 (3)	C10—H10	0.9300
N4—H4	0.8691	O1W—H1WA	0.8501
N11—C2	1.285 (3)	O1W—H1WB	0.8499
N11—C3	2.364 (3)	O2W—H2WA	0.8500
N13—C3	1.299 (3)	O2W—H2WB	0.8500
			
C2—S1—C6	102.11 (11)	C10—C5—S1	150.86 (16)
C2—S1—C5	77.83 (9)	N4—C5—S1	90.18 (13)
C6—S1—C3	76.88 (9)	C9—C5—S1	121.39 (10)
C5—S1—C3	52.39 (6)	C7—C5—S1	62.11 (8)
N11—O12—H12	109.5	C3—C5—S1	64.59 (7)
N13—O14—H14	109.5	C6—C5—C5^i^	85.42 (14)
C3—O14—H14	142.8	C10—C5—C5^i^	90.62 (14)
C3—N4—C5	127.96 (19)	N4—C5—C5^i^	93.28 (14)
C3—N4—H4	113.8	C9—C5—C5^i^	88.25 (10)
C5—N4—H4	118.1	C7—C5—C5^i^	84.92 (10)
C2—N11—O12	110.30 (18)	C3—C5—C5^i^	92.99 (10)
O12—N11—C3	143.99 (15)	S1—C5—C5^i^	87.74 (9)
C3—N13—O14	108.23 (17)	C5—C6—C7	120.0 (2)
N11—C2—C3	117.44 (19)	C5—C6—S1	123.56 (17)
N11—C2—S1	120.86 (17)	C7—C6—S1	116.47 (17)
C3—C2—S1	121.64 (16)	C5—C6—C9	60.51 (13)
N13—C3—N4	123.3 (2)	C7—C6—C9	59.46 (14)
N13—C3—C2	117.02 (19)	S1—C6—C9	175.77 (13)
N4—C3—C2	119.71 (19)	C8—C7—C6	120.4 (2)
N4—C3—O14	85.70 (14)	C6—C7—C9	90.21 (16)
C2—C3—O14	154.59 (16)	C8—C7—C5	90.50 (16)
N13—C3—N11	89.24 (14)	C9—C7—C5	60.34 (10)
N4—C3—N11	146.56 (17)	C8—C7—H7	119.8
O14—C3—N11	126.34 (11)	C6—C7—H7	119.8
N13—C3—C5	149.31 (17)	C9—C7—H7	150.0
C2—C3—C5	93.51 (14)	C5—C7—H7	149.7
O14—C3—C5	111.88 (11)	C7—C8—C9	119.8 (2)
N11—C3—C5	121.44 (11)	C7—C8—H8	120.1
N13—C3—S1	145.97 (16)	C9—C8—H8	120.1
N4—C3—S1	89.42 (13)	C8—C9—C10	120.1 (2)
O14—C3—S1	168.89 (11)	C10—C9—C7	90.00 (16)
N11—C3—S1	60.67 (7)	C8—C9—C5	90.44 (16)
C5—C3—S1	63.02 (7)	C7—C9—C5	60.37 (9)
N13—C3—C9^i^	90.05 (14)	C8—C9—C6	60.40 (14)
N4—C3—C9^i^	88.63 (13)	C10—C9—C6	59.67 (14)
C2—C3—C9^i^	91.67 (14)	C8—C9—C3^i^	80.63 (14)
O14—C3—C9^i^	89.14 (9)	C10—C9—C3^i^	96.59 (15)
N11—C3—C9^i^	99.92 (10)	C7—C9—C3^i^	83.30 (9)
C5—C3—C9^i^	85.70 (8)	C5—C9—C3^i^	92.62 (9)
S1—C3—C9^i^	80.76 (7)	C6—C9—C3^i^	87.82 (8)
C6—C5—C10	118.9 (2)	C8—C9—H9	120.0
C6—C5—N4	122.1 (2)	C10—C9—H9	120.0
C10—C5—N4	119.0 (2)	C7—C9—H9	150.0
C6—C5—C9	89.45 (15)	C5—C9—H9	149.6
N4—C5—C9	148.43 (17)	C6—C9—H9	179.4
C10—C5—C7	88.76 (15)	C3^i^—C9—H9	92.8
N4—C5—C7	152.27 (17)	C9—C10—C5	120.9 (2)
C9—C5—C7	59.29 (10)	C9—C10—H10	119.5
C6—C5—C3	96.54 (15)	C5—C10—H10	119.5
C10—C5—C3	144.53 (17)	H1WA—O1W—H1WB	109.6
C9—C5—C3	173.96 (13)	H2WA—O2W—H2WB	104.7
C7—C5—C3	126.70 (12)		
			
O12—N11—C2—C3	177.69 (18)	C2—S1—C5—C9	170.77 (13)
O12—N11—C2—S1	0.5 (3)	C6—S1—C5—C9	−1.59 (17)
C3—N11—C2—S1	−177.2 (3)	C3—S1—C5—C9	179.04 (14)
C6—S1—C2—N11	−164.88 (19)	C2—S1—C5—C7	171.89 (11)
C5—S1—C2—N11	−168.1 (2)	C6—S1—C5—C7	−0.47 (18)
C3—S1—C2—N11	177.1 (3)	C3—S1—C5—C7	−179.84 (10)
C6—S1—C2—C3	18.0 (2)	C2—S1—C5—C3	−8.27 (10)
C5—S1—C2—C3	14.80 (18)	C6—S1—C5—C3	179.4 (2)
O14—N13—C3—N4	−0.1 (3)	C2—S1—C5—C5^i^	−102.67 (11)
O14—N13—C3—C2	179.59 (18)	C6—S1—C5—C5^i^	85.0 (2)
O14—N13—C3—N11	171.56 (13)	C3—S1—C5—C5^i^	−94.39 (10)
O14—N13—C3—C5	−6.9 (4)	C10—C5—C6—C7	−0.4 (3)
O14—N13—C3—S1	−161.9 (2)	N4—C5—C6—C7	178.8 (2)
O14—N13—C3—C9^i^	−88.52 (14)	C9—C5—C6—C7	−0.5 (2)
C5—N4—C3—N13	−172.2 (2)	C3—C5—C6—C7	−179.75 (18)
C5—N4—C3—C2	8.1 (3)	S1—C5—C6—C7	−179.2 (3)
C5—N4—C3—O14	−172.3 (2)	C5^i^—C5—C6—C7	87.8 (2)
C5—N4—C3—N11	23.0 (4)	C10—C5—C6—S1	178.75 (16)
C5—N4—C3—S1	−2.3 (2)	N4—C5—C6—S1	−2.1 (3)
C5—N4—C3—C9^i^	−83.1 (2)	C9—C5—C6—S1	178.65 (15)
N11—C2—C3—N13	−16.8 (3)	C7—C5—C6—S1	179.2 (3)
S1—C2—C3—N13	160.36 (17)	C3—C5—C6—S1	−0.57 (19)
N11—C2—C3—N4	162.9 (2)	C5^i^—C5—C6—S1	−93.07 (16)
S1—C2—C3—N4	−19.9 (3)	C10—C5—C6—C9	0.10 (16)
N11—C2—C3—O14	−16.2 (5)	N4—C5—C6—C9	179.3 (2)
S1—C2—C3—O14	160.9 (3)	C7—C5—C6—C9	0.5 (2)
S1—C2—C3—N11	177.2 (3)	C3—C5—C6—C9	−179.22 (13)
N11—C2—C3—C5	166.47 (19)	S1—C5—C6—C9	−178.65 (15)
S1—C2—C3—C5	−16.35 (19)	C5^i^—C5—C6—C9	88.29 (10)
N11—C2—C3—S1	−177.2 (3)	C2—S1—C6—C5	−7.6 (2)
N11—C2—C3—C9^i^	−107.7 (2)	C3—S1—C6—C5	0.51 (17)
S1—C2—C3—C9^i^	69.45 (17)	C2—S1—C6—C7	171.56 (17)
N13—O14—C3—N4	179.9 (2)	C5—S1—C6—C7	179.2 (3)
N13—O14—C3—C2	−0.9 (4)	C3—S1—C6—C7	179.71 (18)
N13—O14—C3—N11	−10.50 (17)	C5—C6—C7—C8	0.6 (3)
N13—O14—C3—C5	176.2 (2)	S1—C6—C7—C8	−178.67 (17)
N13—O14—C3—S1	115.7 (6)	C9—C6—C7—C8	0.03 (17)
N13—O14—C3—C9^i^	91.21 (18)	C5—C6—C7—C9	0.5 (2)
C2—N11—C3—N13	165.1 (3)	S1—C6—C7—C9	−178.70 (13)
O12—N11—C3—N13	161.4 (2)	S1—C6—C7—C5	−179.2 (3)
C2—N11—C3—N4	−27.6 (3)	C9—C6—C7—C5	−0.5 (2)
O12—N11—C3—N4	−31.3 (4)	C6—C5—C7—C8	−179.5 (3)
O12—N11—C3—C2	−3.7 (3)	C10—C5—C7—C8	0.11 (18)
C2—N11—C3—O14	171.4 (3)	N4—C5—C7—C8	178.2 (3)
O12—N11—C3—O14	167.7 (2)	C9—C5—C7—C8	−0.13 (13)
C2—N11—C3—C5	−15.9 (2)	C3—C5—C7—C8	−179.20 (15)
O12—N11—C3—C5	−19.6 (3)	S1—C5—C7—C8	−179.02 (15)
C2—N11—C3—S1	1.7 (2)	C5^i^—C5—C7—C8	90.85 (15)
O12—N11—C3—S1	−2.0 (2)	C10—C5—C7—C6	179.6 (3)
C2—N11—C3—C9^i^	75.1 (2)	N4—C5—C7—C6	−2.3 (4)
O12—N11—C3—C9^i^	71.4 (3)	C9—C5—C7—C6	179.4 (2)
C2—S1—C3—N13	−32.4 (3)	C3—C5—C7—C6	0.3 (2)
C6—S1—C3—N13	165.8 (3)	S1—C5—C7—C6	0.50 (19)
C5—S1—C3—N13	166.0 (3)	C5^i^—C5—C7—C6	−89.6 (2)
C2—S1—C3—N4	162.8 (3)	C6—C5—C7—C9	−179.4 (2)
C6—S1—C3—N4	0.88 (13)	C10—C5—C7—C9	0.24 (13)
C5—S1—C3—N4	1.15 (11)	N4—C5—C7—C9	178.4 (4)
C6—S1—C3—C2	−161.9 (2)	C3—C5—C7—C9	−179.07 (15)
C5—S1—C3—C2	−161.6 (2)	S1—C5—C7—C9	−178.89 (10)
C2—S1—C3—O14	−133.4 (7)	C5^i^—C5—C7—C9	90.98 (10)
C6—S1—C3—O14	64.8 (6)	C6—C7—C8—C9	−0.1 (3)
C5—S1—C3—O14	65.0 (6)	C5—C7—C8—C9	0.2 (2)
C2—S1—C3—N11	−1.56 (18)	C7—C8—C9—C10	−0.6 (3)
C6—S1—C3—N11	−163.45 (10)	C7—C8—C9—C5	−0.2 (2)
C5—S1—C3—N11	−163.18 (10)	C7—C8—C9—C6	0.03 (17)
C2—S1—C3—C5	161.6 (2)	C7—C8—C9—C3^i^	−92.8 (2)
C6—S1—C3—C5	−0.27 (9)	C6—C7—C9—C8	180.0 (3)
C2—S1—C3—C9^i^	−108.5 (2)	C5—C7—C9—C8	−179.7 (3)
C6—S1—C3—C9^i^	89.59 (9)	C8—C7—C9—C10	179.5 (3)
C5—S1—C3—C9^i^	89.86 (8)	C6—C7—C9—C10	−0.55 (18)
C3—N4—C5—C6	3.4 (3)	C5—C7—C9—C10	−0.24 (13)
C3—N4—C5—C10	−177.4 (2)	C8—C7—C9—C5	179.7 (3)
C3—N4—C5—C9	−177.9 (2)	C6—C7—C9—C5	−0.31 (12)
C3—N4—C5—C7	4.8 (5)	C8—C7—C9—C6	−180.0 (3)
C3—N4—C5—S1	2.3 (2)	C5—C7—C9—C6	0.31 (12)
C3—N4—C5—C5^i^	90.1 (2)	C8—C7—C9—C3^i^	82.9 (2)
N13—C3—C5—C6	−164.3 (3)	C6—C7—C9—C3^i^	−97.18 (14)
N4—C3—C5—C6	−177.1 (3)	C5—C7—C9—C3^i^	−96.88 (9)
C2—C3—C5—C6	9.93 (18)	C6—C5—C9—C8	0.44 (18)
O14—C3—C5—C6	−168.81 (14)	C10—C5—C9—C8	−179.4 (3)
N11—C3—C5—C6	17.53 (18)	N4—C5—C9—C8	−178.4 (3)
S1—C3—C5—C6	0.34 (11)	C7—C5—C9—C8	0.13 (13)
C9^i^—C3—C5—C6	−81.48 (13)	S1—C5—C9—C8	1.28 (18)
N13—C3—C5—C10	16.7 (5)	C5^i^—C5—C9—C8	−85.00 (15)
N4—C3—C5—C10	3.9 (3)	C6—C5—C9—C10	179.8 (3)
C2—C3—C5—C10	−169.0 (3)	N4—C5—C9—C10	1.0 (3)
O14—C3—C5—C10	12.2 (3)	C7—C5—C9—C10	179.5 (3)
N11—C3—C5—C10	−161.4 (2)	S1—C5—C9—C10	−179.3 (3)
S1—C3—C5—C10	−178.6 (3)	C5^i^—C5—C9—C10	94.4 (2)
C9^i^—C3—C5—C10	99.5 (3)	C6—C5—C9—C7	0.31 (12)
N13—C3—C5—N4	12.8 (3)	C10—C5—C9—C7	−179.5 (3)
C2—C3—C5—N4	−173.0 (3)	N4—C5—C9—C7	−178.5 (3)
O14—C3—C5—N4	8.3 (2)	S1—C5—C9—C7	1.15 (10)
N11—C3—C5—N4	−165.4 (3)	C5^i^—C5—C9—C7	−85.13 (10)
S1—C3—C5—N4	177.4 (3)	C10—C5—C9—C6	−179.8 (3)
C9^i^—C3—C5—N4	95.6 (2)	N4—C5—C9—C6	−178.8 (4)
N13—C3—C5—C7	−164.5 (3)	C7—C5—C9—C6	−0.31 (12)
N4—C3—C5—C7	−177.2 (3)	S1—C5—C9—C6	0.84 (9)
C2—C3—C5—C7	9.77 (19)	C5^i^—C5—C9—C6	−85.44 (14)
O14—C3—C5—C7	−168.97 (12)	C6—C5—C9—C3^i^	81.08 (13)
N11—C3—C5—C7	17.37 (19)	C10—C5—C9—C3^i^	−98.7 (2)
S1—C3—C5—C7	0.18 (11)	N4—C5—C9—C3^i^	−97.8 (3)
C9^i^—C3—C5—C7	−81.64 (13)	C7—C5—C9—C3^i^	80.77 (9)
N13—C3—C5—S1	−164.6 (3)	S1—C5—C9—C3^i^	81.92 (11)
N4—C3—C5—S1	−177.4 (3)	C5^i^—C5—C9—C3^i^	−4.36 (9)
C2—C3—C5—S1	9.60 (11)	C5—C6—C9—C8	−179.5 (2)
O14—C3—C5—S1	−169.15 (11)	C7—C6—C9—C8	−0.03 (17)
N11—C3—C5—S1	17.20 (10)	C5—C6—C9—C10	−0.11 (16)
C9^i^—C3—C5—S1	−81.81 (7)	C7—C6—C9—C10	179.4 (2)
N13—C3—C5—C5^i^	−78.6 (3)	C5—C6—C9—C7	−179.5 (2)
N4—C3—C5—C5^i^	−91.3 (2)	C7—C6—C9—C5	179.5 (2)
C2—C3—C5—C5^i^	95.66 (15)	C5—C6—C9—C3^i^	−99.04 (13)
O14—C3—C5—C5^i^	−83.09 (12)	C7—C6—C9—C3^i^	80.43 (14)
N11—C3—C5—C5^i^	103.26 (13)	C8—C9—C10—C5	0.7 (3)
S1—C3—C5—C5^i^	86.06 (9)	C7—C9—C10—C5	0.4 (2)
C9^i^—C3—C5—C5^i^	4.25 (9)	C6—C9—C10—C5	0.11 (16)
C2—S1—C5—C6	172.4 (2)	C3^i^—C9—C10—C5	83.7 (2)
C3—S1—C5—C6	−179.4 (2)	C6—C5—C10—C9	−0.2 (3)
C2—S1—C5—C10	170.1 (3)	N4—C5—C10—C9	−179.4 (2)
C6—S1—C5—C10	−2.2 (3)	C7—C5—C10—C9	−0.4 (2)
C3—S1—C5—C10	178.4 (3)	C3—C5—C10—C9	178.6 (2)
C2—S1—C5—N4	−9.39 (13)	S1—C5—C10—C9	1.1 (4)
C6—S1—C5—N4	178.2 (3)	C5^i^—C5—C10—C9	−85.3 (2)
C3—S1—C5—N4	−1.12 (11)		

Symmetry codes: (i) −*x*+1, −*y*+2, −*z*+1.

### Hydrogen-bond geometry (Å, °)

**Table:**

*D*—H···*A*	*D*—H	H···*A*	*D*···*A*	*D*—H···*A*
O1W—H1WA···N11^ii^	0.85	2.11	2.931 (2)	162
O1W—H1WA···N13^ii^	0.85	2.62	3.225 (2)	129
O1W—H1WB···O2W^ii^	0.85	2.00	2.845 (2)	171
O2W—H2WA···N13^iii^	0.85	2.02	2.853 (3)	168
N4—H4···O14	0.87	2.08	2.493 (2)	108
O2W—H2WB···O1W	0.85	1.94	2.780 (2)	171
O12—H12···O2W^iv^	0.82	1.89	2.699 (2)	171
O14—H14···O1W	0.82	1.85	2.673 (2)	179

Symmetry codes: (ii) *x*+1/2, *y*, −*z*+1/2; (iii) −*x*+1, *y*+1/2, −*z*+1/2; (iv) −*x*+1/2, *y*−1/2, *z*.

## References

[bb1] Bruker (1998). *SMART* and *SAINT-Plus* Bruker AXS Inc., Madison, Wisconsin, USA.

[bb2] Ghiasvand, A. R., Ghaderi, R. & Kakanejadifard, A. (2004). *Talanta*, **62**, 287–292. 10.1016/j.talanta.2003.07.011 18969293

[bb3] Ghiasvand, A. R., Shadabi, S., Kakanejadifard, A. & Khajehkolaki, A. (2005). *Bull. Korean Chem. Soc.* **26**, 781–785.

[bb4] Gok, Y. & Kantekin, H. (1997). *Polyhedron*, **16**, 2413–2420.

[bb5] Hashemi, P., Rahmani, Z., Kakanejadifard, A. & Niknam, E. (2006). *Anal. Sci.* **21**, 1297–1301. 10.2116/analsci.21.1297 16317897

[bb6] Hughes, M. N. (1981). *The Inorganic Chemistry of Biological Processes* New York: Wiley.

[bb7] Jones, M. E. B., Thornton, D. A. & Webb, R. F. (1961). *Makromol. Chem.* **49**, 62–66.

[bb8] Kakanejadifard, A. & Amani, V. (2008). *Acta Cryst.* E**64**, o1512. 10.1107/S1600536808021570 PMC2962138 21203220

[bb9] Kakanejadifard, A. & Niknam, E. (2006). *Pol. J. Chem.* **80**, 1645–1649.

[bb10] Kakanejadifard, A., Niknam, E., Ranjbar, B. & Naderi-Manesh, H. (2007). *Synth. Commun.* **37**, 2753–2756.

[bb11] Kakanejadifard, A., Niknam, E. & Zabardasti, A. (2007). *J. Coord. Chem.* **60**, 677–681.

[bb12] Kakanejadifard, A., Saniei, A., Delfani, F., Farnia, M. & Najafi, G. R. (2007). *J. Heterocycl. Chem.* **44**, 717–718.

[bb13] Schrauzer, G. N. & Kohnle, J. (1964). *Chem. Ber.* **97**, 3056–3063.

[bb14] Sheldrick, G. M. (1998). *SADABS* Bruker AXS Inc., Madison, Wisconsin, USA.

[bb15] Sheldrick, G. M. (2008). *Acta Cryst.* A**64**, 112–122. 10.1107/S0108767307043930 18156677

[bb16] Yari, A., Azizi, S. & Kakanejadifard, A. (2006). *Sens. Actuators B*, **119**, 167–173.

